# Estimation of Time-Varying Coherence Amongst Synergistic Muscles During Wrist Movements

**DOI:** 10.3389/fnins.2018.00537

**Published:** 2018-08-07

**Authors:** Guiting Hu, Wenjuan Yang, Xiaoling Chen, Wenjing Qi, Xinxin Li, Yihao Du, Ping Xie

**Affiliations:** Key Lab of Measurement Technology and Instrumentation of Hebei Province, Institute of Electric Engineering, Yanshan University, Qinhuangdao, China

**Keywords:** EMG, muscle synergy, intermuscular coherence, non-negative matrix factorization, time-frequency analysis

## Abstract

The central nervous system (CNS) controls the limb movement by modulating multiple skeletal muscles with synergistic modules and neural oscillations with different frequencies between the activated muscles. Several researchers have found intermuscular coherence existing within the synergistic muscle pairs, and pointed out that the intermuscular synchronization existed when functional forces were generated. However, few studies involved the time-varying characteristics of the intermuscular coherence in each synergy module though all activated muscles keep in a dynamic and varying process. Therefore, this study aims to explore the time-varying coherence amongst synergistic muscles during movements based on the combination of the non-negative matrix factorization (NMF) method and the time-frequency coherence (TFC) method. We applied these methods into the electromyogram (EMG) signals recorded from eight muscles involved in the sequence of the wrist movements [wrist flexion (WF), wrist flexion transmission to wrist extension (MC) and wrist extension (WE)] in 12 healthy people. The results showed three synergistic flexor pairs (FCR-PL, FCR-FDS, and PL-FDS) in the WF stage and three extensor pairs (ECU-ECR, ECU-B, and ECR-B) in both MC and WE stages. Further analysis showed intermuscular coherence between each pairwise synergistic muscles. The intermuscular coherence between the flexor muscle pairs was mainly observed in the beta band (15–35 Hz) during the WF stage, and that amongst the extensor muscle pairs was also observed in the beta band during the WE stage. However, the intermuscular coherence between the extensor muscle pairs mainly on gamma band during the MC stage. Additionally, compared to the flexor muscle pairs, the intermuscular coherence of the extensor muscle pairs were lower in the WF stage, and higher in both MC and WE stages. These results demonstrated the time-varying mechanisms of the synergistic modulation and synchronous oscillation in motor-control system. This study contributes to expanded researches for motor control.

## Introduction

The central nervous system (CNS) regulates the movement by modulating multiple skeletal muscles (Gottlieb, 1998). In this process, modular structures are often used to organize and coordinate multiple degree-of-freedom change among muscles (Carpenter, 1968; d'Avella et al., 2003), and different movement behaviors are formed by arranging certain synergistic modules (Ting and Mckay, 2007). Therefore, it is of interest to understand how muscle activation patterns are employed to activate different motor functions (d'Avella and Bizzi, 2005).

Previous studies have pointed out that the CNS controls the muscles by dividing the enrolled muscles into various modules (De et al., 2015), and these modules are selectively activated at certain points. In each module, the muscles can be activated in synergy to varying degrees (Tang et al., 2017). For every synergistic event, the output signal of the nervous system is transformed to motor activation at the task-level (Geyer and Herr, 2010). To explore the synergy among muscle groups in different modules, the non-negative matrix factorization (NMF) (Lee, 1999) method was introduced. Considering that the NMF method can break the data into a target matrix without any negative, large studies have applied this method to analyze the muscle activation during pedaling (De et al., 2015), walking (Haghpanah et al., 2017), and elbow movement (Tang et al., 2017) and so on. Although muscle synergy can reflect the relationship of the combination and coordination among the multiple muscles and provide insight into the overall muscular activity (d'Avella et al., 2013), it has limitation in exploring the synchronous oscillations among muscles that play an important role in motor-control system (Li et al., 2016).

Recent studies showed that the intermuscular coherence between two muscles can reveal the neural oscillations with different frequencies (Marchis et al., 2012). Many studies found that intermuscular coherence presented at specific frequencies during different motor task (de Vries et al., 2016; Laine and Valerocuevas, 2017). Chakarov, et al. found that the corticomuscular synchronization occurred in beta (15–30 Hz) and gamma range (30–45 Hz) during isometric compensation of static and dynamic low-level forces, respectively (Chakarov et al., 2009). Kristeva R et al. found that the beta-range cortical motor spectral power and corticomuscular coherence may promote effective corticospinal interaction (Kristeva and Patino, 2007). However, intermuscular coherence only can reflect the oscillations between two analyzed muscles, but ignore the inherently synergistic effect in motor control. Farmer proposed the motor binding hypothesis that synergistic muscles were coupled by synchronous neural oscillation (Farmer, 1998). A few researches provide a novel perspective by combining the muscle synergy and intermuscular coherence to analyze EMG signals (De et al., 2015; Li et al., 2016). They analyzed the intermuscular coherence between each muscle pair with or without synergy based on the NMF and coherence methods. This study demonstrates that the presence of intermuscular synchronization only in function force producing (De et al., 2015). In addition, the coherence method applied in above researches only can analyze the rhythm characteristics in the frequency domain. Considering that the complexity and dynamics in the motor-control system result in the time-varying and non-stationary characteristics of the EMG signals (Subasi and Kiymik, 2010), the coherence method is lack of describing the dynamic time-varying characteristics. The time-frequency coherence (TFC) method could describe time-varying characteristics. Many studies have used the TFC method to investigate the role of neural oscillation and pointed out that intermuscular synchronization presented in different bands during different motor tasks (Boonstra et al., 2009; Jiang and Mahadevan, 2011). However, there was no similar analysis about the time-varying coherence within the synergy muscles. Above all, it is necessary to combine NMF and TFC to analyze the time-varying synchronization of muscle synergy and intermuscular coupling.

To explore the time-varying coherence characteristic within the synergistic muscles, we applied the NMF and TFC methods to analyze the electromyogram (EMG) signals recorded from eight muscles involved in conversion between wrist flexion (WF) and wrist extension (WE) movements in 12 healthy people. Firstly, we extracted the synergistic muscle pairs. And then, the TFC method was used to perform time-frequency decomposition to analyze the time-varying coherence in synergistic pairs. This study is useful for detecting functional connections between muscles and provides a basis for studying the control mechanisms in human movement.

## Materials and methods

### Subjects

Twelve healthy and right-handed subjects (aged 23 ± 2 years) without any previous history of neural or physiological disorders were participated in this experiment. Before the experiments, each subject completed the Oldfield questionnaire (Oldfield, 1971) and signed an informed consent form. The experiment was approved by the ethical review board of Yanshan University and was performed in accordance with the Declaration of Helsinki. To avoid the influence of fatigue, all subjects were in a good state of mind, and they had undergone no recent strenuous exercise.

### Experimental protocol and data recording

**Figure 1:**
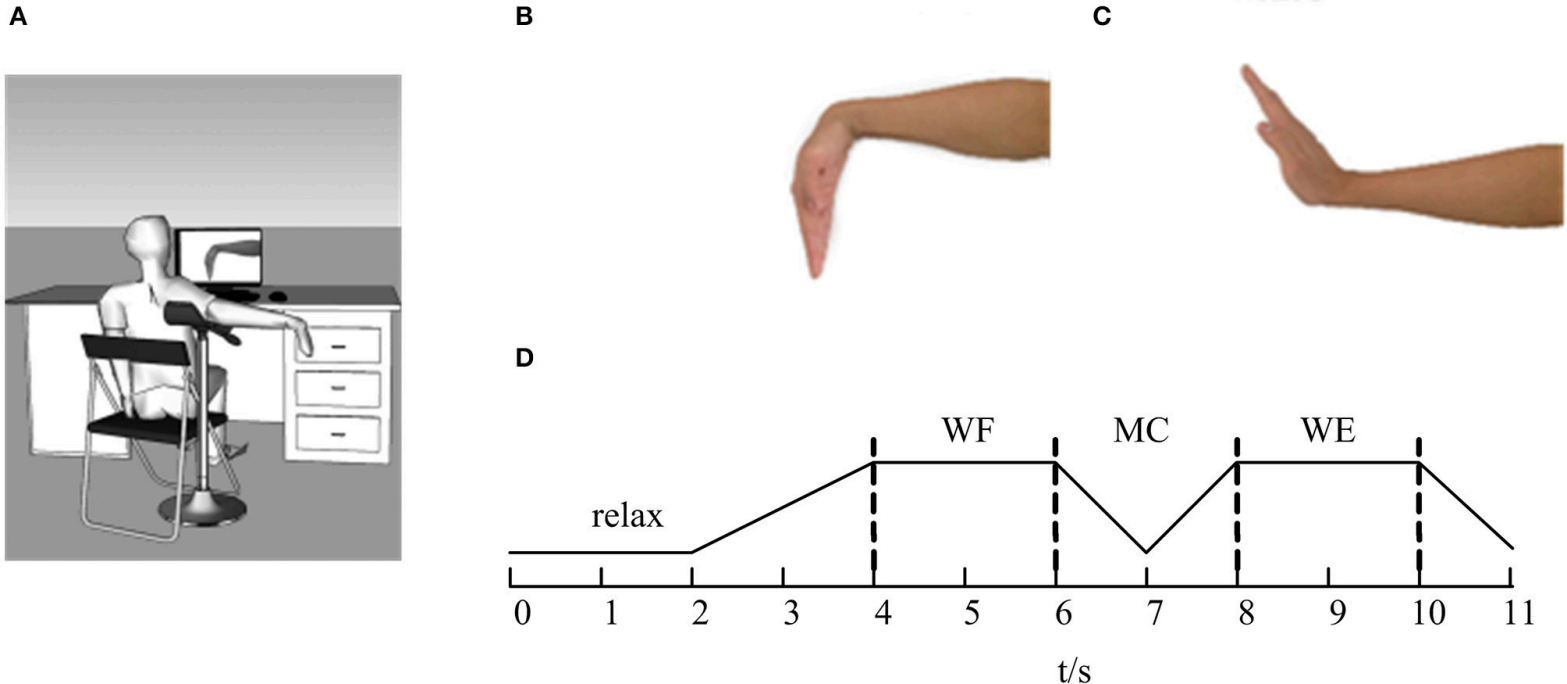
The diagram of the experiment. **(A)** The sketch of the experiment. **(B)** Wrist flexion (WF). **(C)** Wrist extension (WE). **(D)** The flow of the task. The line represents the maintenance phase, and the dotted lines represent the state transitions.

The experiments were performed in a closed, soundproof room to avoid the noise interference. Before the EMG signals were collected, all subjects were asked to sit comfortably in front of a computer screen. Figure 1 showed the diagram of this experiment. Before the task, the subject was asked to adjust the shoulder and elbow to a horizontal position at a 90° angle with the arm on the upper limb bracket (Figure 1A). After that, the subject performed the task according to the target movement (Figures 1B,C) displayed on the computer screen.

During the experiment, the subjects were instructed to concentrate on the computer screen. As Figure 1D was shown, the whole task mainly involved four parts. In each trial, the subjects began in a relaxed state for 2 s. When the WF task appeared on the computer screen, the subjects began to perform the WF movement and maintained a steady-state WF movement for 2 s until the WE instruction appeared on the screen. Next, the subjects gradually performed the dynamic process of transitioning from the WF to the WE for 2 s (shown as MC stage in Figure 1D) and maintained a steady-state WE movement for 2 s until the WE instruction disappeared. Finally, the subjects returned to the resting state and maintained a relaxed state for 1 s until the ending instruction appeared. Ten trials were carried out for each subject, and each trial lasted 11 s. Each subject rested for 10 s after each trial to avoid the influence of muscle fatigue. Before the experiment, we measured the maximum voluntary contraction (MVC) for both WF and WE movements with a force sensor pasted on the wrist. All subjects performed the WF and WE task with the MVC.

**Figure 2:**
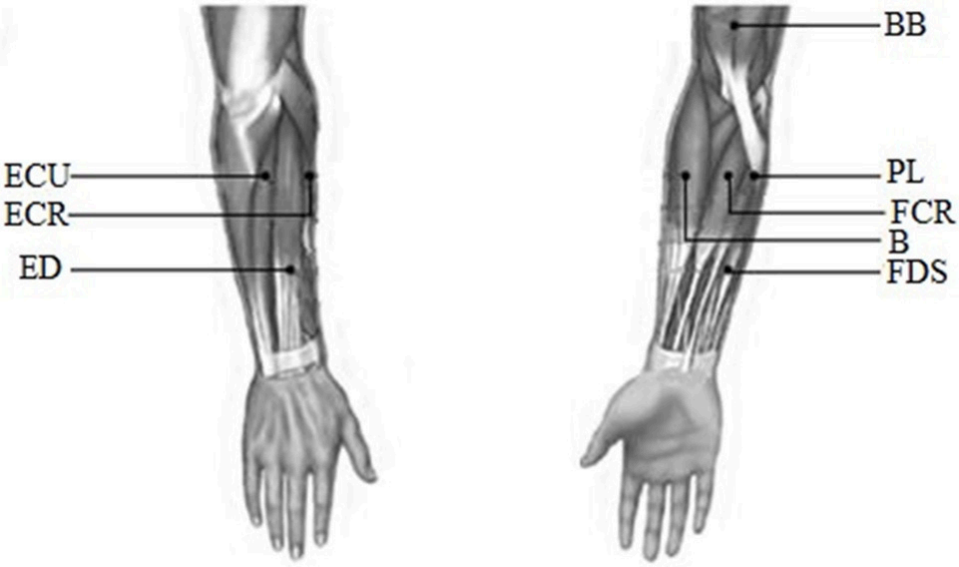
The diagram of the electrode position.

EMG signals were recorded from the following eight muscles of the upper limb: biceps brachii (BB), brachioradialis (B), flexor carpi radialis (FCR), palmaris longus (PL), extensor carpi radialis (ECR), extensor digitorum (ED), extensor carpi ulnaris (ECU), and flexor digitorum superficialis (FDS). All these muscles were considered to be the main muscle groups involved in the wrist movements. EMG signals were recorded with a Trigno™ Wireless EMG system (Delsys Inc., USA). The detail about this system was described in Appendix (Supplementary Material). The recorded EMG data were band-pass filtered (0–450 Hz) with a power frequency suppression device in this system. Before the experiment, the surface of the skin was cleaned with alcohol to remove grease. Finally, each Trigno sensor was placed on the muscles along the direction of the muscle fibers. The electrode position was as shown in Figure 2.

### Data processing

#### Data preprocessing

To extract the surface EMG signals profiles, the raw EMG signals need to be preprocessed. First, we removed the data segments with serious hand trembling or slow responses. Then all EMG signals were downsampled to 500 Hz. Finally, the signals were filtered with a 5–150 Hz bandpass filter and full-wave rectified to obtain the signal envelope for further analysis.

#### Non-negative matrix factorization

**Figure 3:**
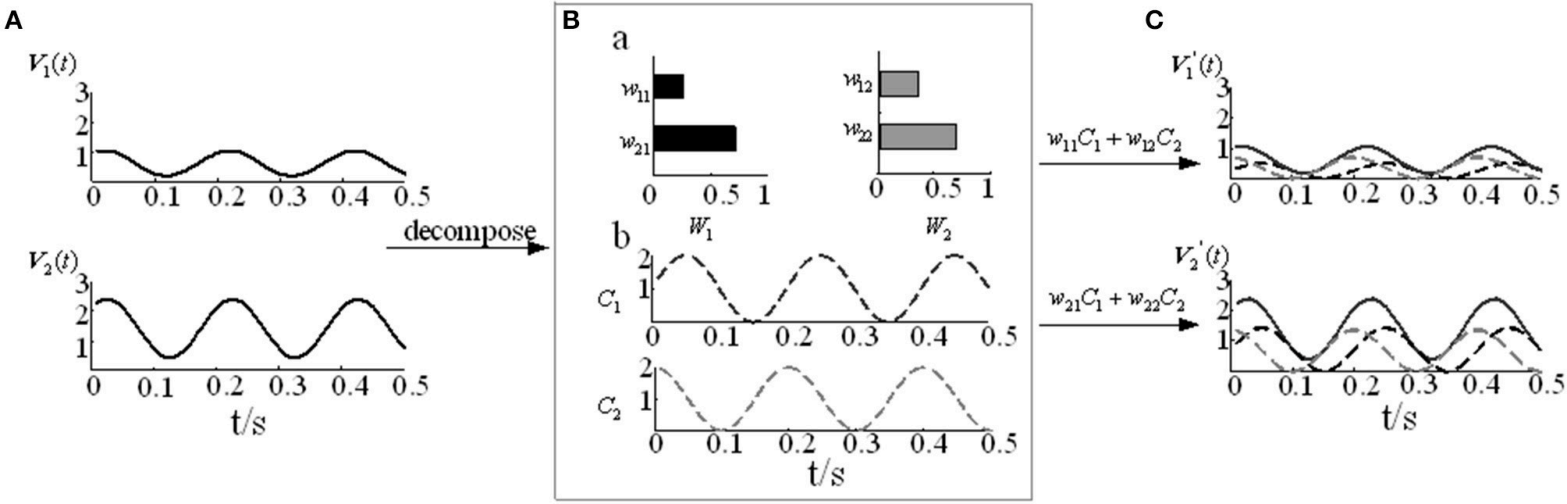
The NMF decomposition model. **(A)** Two-channel EMG signals *V*_1_ and *V*_2_. **(B)** The muscle activation model. **(B-a)** Muscle synergy vector matrices *W*_1_ and *W*_2_. **(B-b)** Time-varying coefficients *C*_1_ and *C*_2_. **(C)** Reconstruction signals $V\prime_{1}$ and $V\prime_{2}$.

To analyze the synergy among the multi-channel EMG signals, muscle synergies were extracted by applying the NMF method to the preprocessed EMG signals. The EMG signal is regarded as *V*_*mn*_ matrix, which can be considered a muscle activation model. This matrix can be seen as a linear combination of the muscle synergy vector matrix *W* and the time-varying coefficient *C*. The muscle activation model can be expressed as the following formula:

(1) $$\begin{array}{l} V_{mn}\approx {\left(WC\right)}_{mn}=\overset{k}{\underset{i=1}{\sum }}W_{mi}C_{in}={V^{\prime }}_{mn} \end{array}$$

where *m* represents the number of signals, *n* represents the number of sampling points, *k* represents the number of muscle synergy, *V*_*mn*_ represents the numerical matrix of m channels EMG signals, and *W* is the muscle synergy vector matrix, which represents the relative weight of each muscle in the *i-th* muscle synergy. *C* is the time-varying coefficient, which represents how the *i-th* synergy matrices are modulated at time *t* and reflects the contribution of each muscle synergy to the movement. ${V^{\prime }}_{mn}$ contains the reconstructed EMG signals based on the decomposition model. The detailed NMF modular decomposition model is shown in Figure 3.Where *V* contains the constructed two-channel EMG signals (Figure 3A). In Figure 3B, when the number of synergy columns *k* is 2, *V* is decomposed into the muscle synergy vector matrix *W*_22_ and the time-varying coefficient *C*_2*n*_. By reconstructing the two decomposed matrices, we can obtain the reconstructed matrix *V*′ (Figure 3C).

The number of synergy modules *k* generally depends on the value of the variability accounted for (VAF) (Kattla and Lowery, 2010) before applying NMF. The VAF is defined as follows:

(2) $$\begin{array}{l} VAF=1-\frac{RSS}{TSS}=1-\frac{\sum {\left(V-V^{\prime }\right)}^{2}}{\sum {\left(V\right)}^{2}} \end{array}$$

where *RSS* is the residual sum of squares, and *TSS* is the total sum of squares.

A previous study (Clark et al., 2010) found that when the number of synergy modules was small, it was a superposition of many modules. To reconstruct the signals in detail and retain the useful information of the original signals, *k* was chosen as the smallest number able to explain at least 92% of the VAF. Additional synergies were not extracted if the synergy modules with the lowest VAF value showed an increase in VAF of < 1% when extracting *k*+1 synergies.

#### Time-frequency coherence

To analyze the intermuscular coherence amongst synergistic muscle pairs, we applied the short-time Fourier transform (STFT) (Mehrkanoon et al., 2013) to estimate the time-frequency distribution of signals, which has been used for spectral decomposition due to the steady time-frequency resolution.

Firstly, the two channels EMG signals *x*(*t*) and *y*(*t*)(*t* = 1, 2, ⋯ , *n*) were first divided into *L* segments of equivalent length by multiplying an appropriate weighting window *w*(*t*) (Dudkina and Tverskaia, 2007), respectively, which was a unit power Hamming window. Where *n* is the total length of the series *x*(*t*) or *y*(*t*). The STFT formulas with the window of signals are as follows:

(3) $$\begin{array}{l} X_{l}\left(f\right)=\frac{1}{T}\overset{T}{\underset{t=1}{\sum }}x_{l}\left(t\right)w\left(t\right)e^{-j2\frac{\pi }{T}ft},\left(l=1,2,\cdots \;,L\right) \end{array}$$

(4) $$\begin{array}{l} Y_{l}\left(f\right)=\frac{1}{T}\overset{T}{\underset{t=1}{\sum }}y_{l}\left(t\right)w\left(t\right)e^{-j2\frac{\pi }{T}ft},\left(l=1,2,\cdots \;,L\right) \end{array}$$

where *X*_*l*_(*f*) and *Y*_*l*_(*f*) are the time-frequency domain representation of the series *x*_*l*_(*t*) and *y*_*l*_(*t*), respectively. The series *x*_*l*_(*t*) = *x*[*l*(*T*−*q*)+*t*] and *y*_*l*_(*t*) = *y*[*l*(*T*−*q*)+*t*] are two channels of the EMG signals in the *l-th* window, respectively. *T* is the width of a weighting window function *w*(*t*) and *q* is the overlapping samples. In our study, we set *T* = 500 and *q* = 450.

Then TFC is computed by smoothing the cross- and auto-spectra using a smoothing kernel specified by identical convolution operator *v*(*t*). The expression of the TFC between the two EMG signals is as follows:

(5) $$\begin{array}{l} C_{xy}^{2}\left(l,f\right)=\frac{{\left|\overset{\wedge }{p_{xy}}\left[l,f\right]⊗v\left[t\right]\right|}^{2}}{\left\{{\left|\overset{\wedge }{p_{xx}}\left[l,f\right]\right|}^{2}⊗v\left[t\right]\right\}\left\{{\left|\overset{\wedge }{p_{yy}}\left[l,f\right]\right|}^{2}⊗v\left[t\right]\right\}} \end{array}$$

where $\overset{\wedge }{p_{xx}}\left[l,f\right]={\left|X_{l}\left(f\right)\right|}^{2}$ and $\overset{\wedge }{p_{yy}}\left[l,f\right]={\left|Y_{l}\left(f\right)\right|}^{2}$ are the auto-spectra of the *x*(*t*) and *y*(*t*), respectively. $\overset{\wedge }{p_{xy}}\left[l,f\right]=X_{l}\left(f\right)Y_{l}^{*}\left(f\right)$ is the cross-spectra of the *x*(*t*) and *y*(*t*). The asterisk * represents a complex conjugate. The *v*(*t*) is a convolution operator, which is a convolution matrix obtained by a 6 * 1 Gaussian window multiplying a 1 * 80 Gaussian window. The ⊗ is the convolution symbol. To assume values in the range of [0–1], we applied identical convolution operators for PSD and CSD. As a result, all the coherence values of each time *t* and frequency *f* ranged from 0 to 1. When $C_{xy}^{2}=0$, *x* and *y* are irrelevant; when $C_{xy}^{2}=1$, there are strongest coupling between the *x* and *y* signals.

In subsequent analysis, considering that the number of data collected for each subject, we calculated the grand average TFC values across repeated trials to present the results intuitively for each subject:

(6) $$\begin{array}{l} \bar{C}=\frac{1}{r}\overset{r}{\underset{r=1}{\sum }}C_{i}^{2}\left(l,f\right) \end{array}$$

where *r* is the number of trials and $C_{i}^{2}\left(l,f\right)=C_{xy}^{2}\left(l,f\right)$.

To assess the statistical significance of the TFC values as *C*_*xy*_(*l, f*) at time-frequency scale, we used the surrogate data method by randomizing the phase of the original data, which can only change the phase information without changing the time domain amplitude and frequency domain power spectrum value. For surrogate data, we also calculated the mean TFC values as $C_{xy}^{\prime }\left(l,f\right)$ across repeated trials. In our study, we calculated the difference values by the *C*_*xy*_(*l, f*) values subtracting the $C_{xy}^{\prime }\left(l,f\right)$ values named *DC*_*xy*_(*l, f*). If the difference value was positive, we consider that the coupling strength was significant, which can represent strong functional connection between two EMG signals at a specific time and frequency. And we defined as zero if the difference value was negative.

### Statistical analysis

To quantitatively describe the changes of the intermuscular coherence amongst the synergistic muscles during movement, we also defined the TFC area at a specific time period based on the significant coherence area, termed as *A*_*Z*_. The *A*_*Z*_ in special frequency band (*f*_1_~ *f*_2_) and time range (*t*_1_~ *t*_2_) can be expressed as follows:

(7) $$A_{Z}=\sum_{l=t_{1}}^{t_{2}}\Delta l\sum_{f=f_{1}}^{f_{2}}\Delta f[C_{xy}(l,f)-C_{xy}\prime (l,f)]$$

where Δ*l* represents the time resolution, and Δ*f* represents the frequency resolution.

After that, we performed three-way repeated measures analysis of variance (ANOVA) with synergy muscles, stage and frequency band within-subject factors, and the significant area *A*_*Z*_ values as the dependent variable. Greenhouse-Geisser was used to correct the degree of freedom. In this study, an alpha of *p* < 0.05 was considered significant. SPSS 19.0 for windows (SPSS Inc., Chicago, IL, USA) was used for all statistical analysis.

## Results

### Muscles synergy analysis

**Figure 4:**
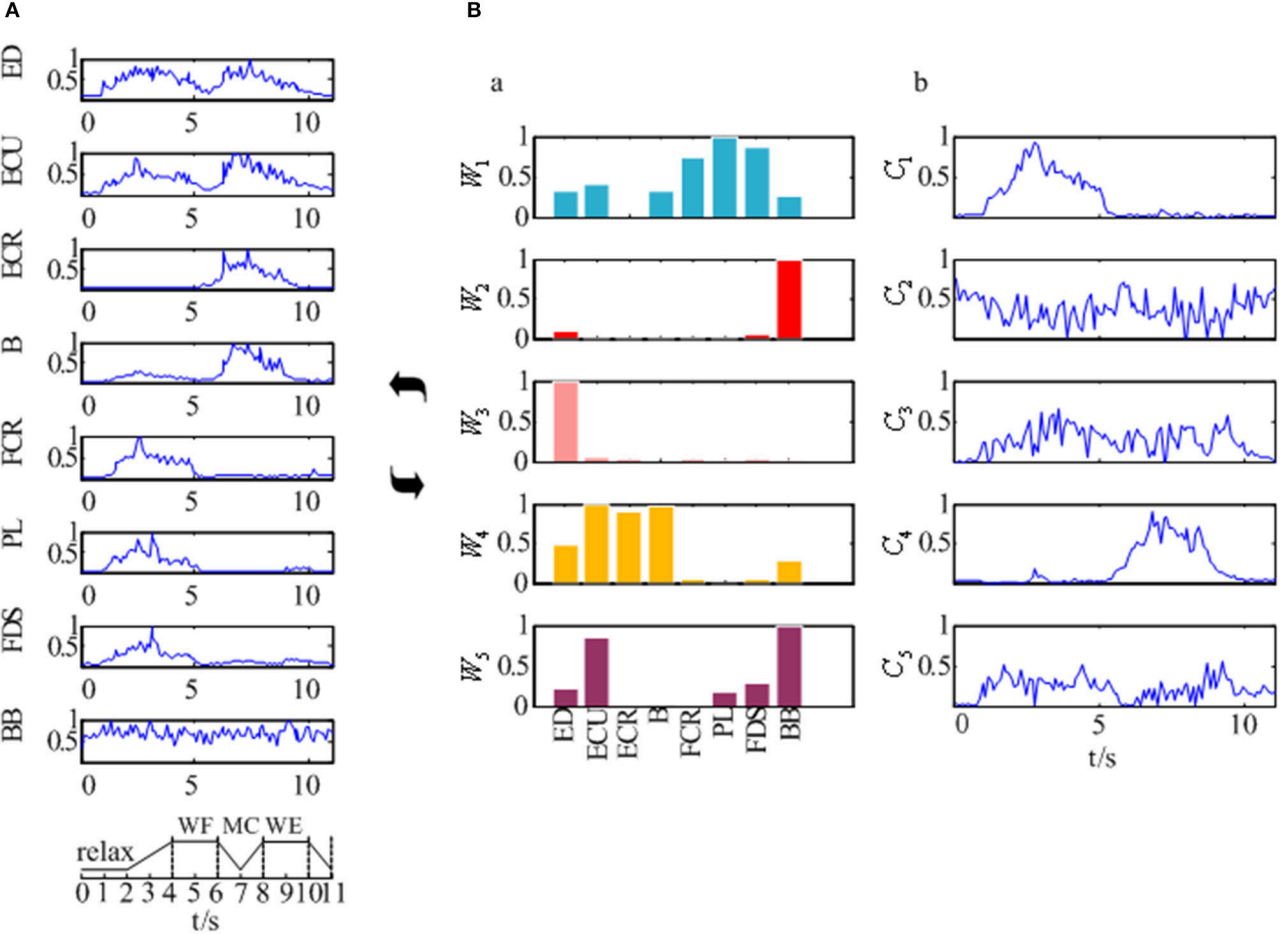
The synergy muscle analysis based on the NMF. **(A)** The preprocessed EMG signals of the eight muscles. **(B)** Muscle activation model based on the NMF. **(B-a)** Muscle synergy matrix. **(B-b)** Time-varying synergy coefficient.

The EMG signals of the 8 muscles were collected according to the experimental process described in section Experimental Protocol and Data Recording. Figure 4A showed the preprocessed EMG signals in 8 muscles for one subject which was selected randomly from all 12 subjects. As Figure 4A was shown, although the EMG signals of these muscles differed along the sequence of the wrist movement, there was some certain regularity in the whole. The EMG signals of the PL, FDS, and FCR muscles showed a high peak during the WF stage, while the EMG signals of the B and ECR muscles showed a high peak during the WE stage. In addition, there were two peaks in the EMG signals of the ED and ECU muscles, although the second peak was higher compared to the first one. However, the EMG signal of the BB muscle showed no obvious changes during the movement.

**Figure 5:**
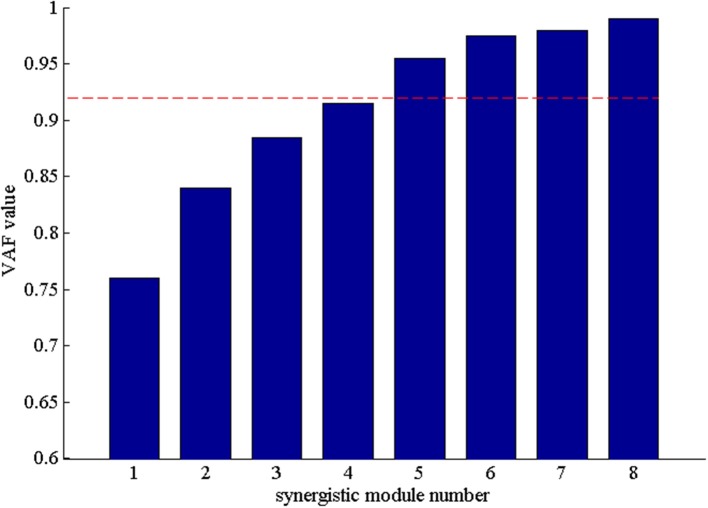
The VAF values with the number of the synergy modules increasing. The red dash denoted that VAF value is 0.92.

After that, we extracted the muscle synergies from the EMG data in eight muscles involved into the sequence of the wrist movement for all subjects to find the changes of the synergy muscles during the whole movement. Figure 4B showed the grand average values of the 10 trials for the same subject. Before the analysis of the synergy, we need make a decision about the number of the synergy modules. Figure 5 showed the result for one subject. As Figure 5 was shown, when the number of synergy modules was 5, the VAF values satisfied the condition as described in our study. And the VAF values for other subjects were listed in Table 1. We can see that most of the VAF values could satisfy the condition when the synergy module was 5. Above all, we selected the number of synergy modules as 5 to determine the synergy structure for subsequent study.

**Table 1:** The VAF values under different numbers of the synergy module for all subjects.

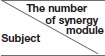	**1**	**2**	**3**	**4**	**5**	**6**	**7**	**8**
S1	0.76	0.83	0.87	0.92	0.96	0.98	0.96	0.98
S2	0.75	0.82	0.86	0.93	0.97	0.96	0.96	0.97
S3	0.75	0.84	0.87	0.90	0.94	0.97	0.97	0.98
S4	0.74	0.83	0.86	0.92	0.96	0.95	0.97	0.97
S5	0.75	0.82	0.88	0.91	0.95	0.96	0.97	0.98
S6	0.76	0.81	0.87	0.93	0.95	0.97	0.97	0.97
S7	0.78	0.85	0.86	0.90	0.96	0.96	0.96	0.98
S8	0.74	0.86	0.89	0.91	0.97	0.99	0.97	0.97
S9	0.76	0.85	0.88	0.93	0.96	0.97	0.97	0.98
S10	0.72	0.85	0.89	0.93	0.94	0.97	0.97	0.97
S11	0.71	0.82	0.86	0.91	0.94	0.98	0.97	0.97
S12	0.71	0.85	0.87	0.90	0.95	0.98	0.96	0.97

Additionally, Figure 4B showed us the results of the synergistic muscles. According to the number of synergy modules in Figure 5, the column in Figure 4B-a represented the muscle synergy vector matrix *W* (*W*_1_~*W*_5_), and the column in Figure 4B-b represented the time-varying coefficient *C* (*C*_1_~*C*_5_). To exhibit the weight value of the active muscle based on the muscle synergy vector matrix *W*, the *W* value was normalized in the range [0–1]. The muscles were considered as active muscles if their weight values were higher than 0.5. As Figure 4B-b was shown, only the coefficient *C*_1_ and *C*_4_ has a peak value over their own weight values. In additionally, the peak of the coefficient *C*_1_ lied at the WF stage and the peak of the coefficient *C*_4_ lied at the MC and WE stages. Therefore, the module *W*_1_ and *W*_4_ played an important role in the synergy during the whole wrist movement. As Figure 4B-a was shown, three muscles acted in the module *W*_1_: FCR, PL, and FDS muscles, which resulted in three pairs of synergy muscles (FCR-PL, FCR-FDS, and PL-FDS). Similarly, three muscles also activated in the module *W*_4_: ECU, ECR, and B muscles, which led to three pairs of synergy muscles (ECU-ECR, ECU-B, and ECR-B). Compared to Figure 4A, we can see that the synergistic level of the muscles in each module was almost consistent with the activity level of their own during the whole movement. As a result, we could conclude that three flexor pairs (FCR-PL, FCR-FDS, and PL-FDS) were synergistic in the WF stage and three extensor pairs (ECU-ECR, ECU-B, and ECR-B) were synergetic in both MC and WE stages. After that, we also summarized the synergy results for other subjects in Table 2. It showed the similar results.

**Table 2:** The synergistic muscles of all subjects.

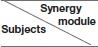	***W*_1_**	***W*_2_**	***W*_3_**	***W*_4_**	***W*_5_**
S1	FCR,PL,FDS	BB	ED	ECU,ECR, B	ECU,BB
S2	BB	FCR,PL,FDS	ED,BB	ECU,ECR,B,ED	
S3	FCR,PL,FDS,BB	ED,ECU	BB	ECU,ECR,B	ECR,BB
S4	FDS,BB	ECU,ECR,B	FCR,PL,FDS	ED	BB
S5	ECU,ECR,B,BB	BB	ED	FCR,PL,FDS	FDS,BB
S6	FCR,PL,FDS,BB	ECU,ECR,B	FDS, PL	,BB	
S7	BB	FCR,PL,FDS	ECU,ECR,B	ED	ECU,BB
S8	FCR,PL,FDS	ECU,ECR,B	BB	ECU	FDS,BB
S9	FCR,PL,FDS,BB	ED,ECU	BB	ECU,ECR,B	ECR,BB
S10	FCR,PL,FDS	BB	ED	ECU,ECR, B	
S11	FDS,BB	ECU,ECR,B	FCR,PL,FDS	ED	BB
S12	ECU,ECR,B,BB	BB	ED	FCR,PL,FDS	FDS,BB

### Time-frequency coherence analysis within synergistic muscle pairs

**Figure 6:**
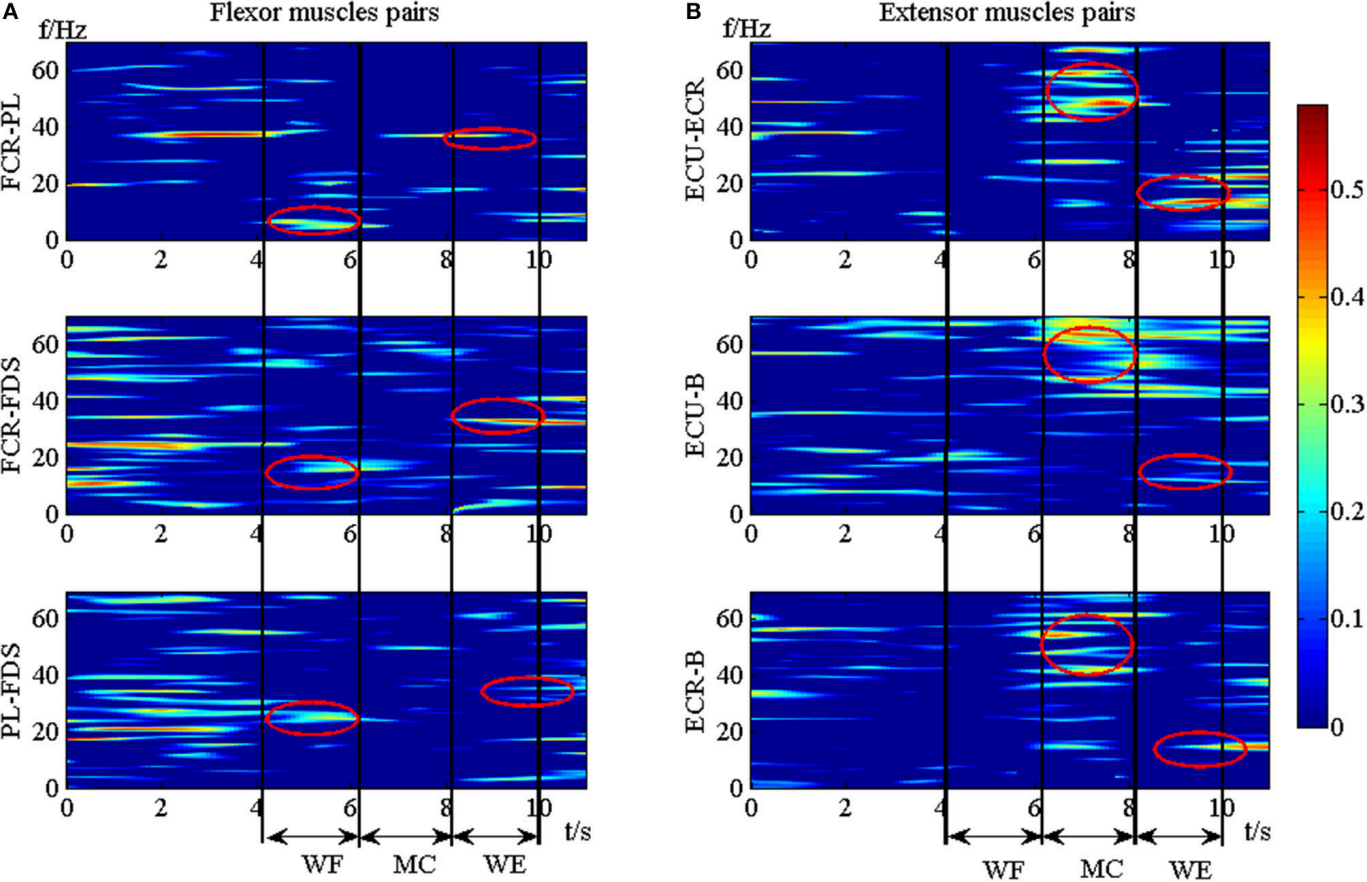
The TFC values of the synergistic muscle pairs. **(A)** The TFC values for flexor muscle pairs. **(B)** The TFC values for extensor muscle pairs.

To explore the TFC within the synergistic muscle pairs, we chose six synergistic muscle pairs to further analyze the intermuscular coherence according to Figure 4. Figure 6 showed the *DC*_*xy*_(*l, f*) values within synergistic muscle pairs. Figure 6A represented the *DC*_*xy*_(*l, f*) value within the flexor muscle pairs FCR-PL, FCR-FDS, and PL-FDS, and Figure 6B was for extensor muscle pairs ECU-ECR, ECU-B, and ECR-B. As Figure 6A was shown, the *DC*_*xy*_(*l, f*) values of the FCR-FDS and PL-FDS pairs were high in the beta band (15–35 Hz) and that of the FCR-PL pair was higher in the alpha band (8–15 Hz) during WF stage. Additionally, there was also weaker coherence in the gamma band (35–60 Hz) during the WE stage than during the WF stage. However, there was small value during the MC stage. Compared to the results of the flexor muscle pairs, Figure 6B showed that the *DC*_*xy*_(*l, f*) value of the extensor muscle pairs were higher in the gamma band during the MC stage, while were higher in the beta band during the WE stage. However, there was no significant coherence during the WF stage. In short, the *DC*_*xy*_(*l, f*) values was higher at the beta band during the WE and WF stage, and gamma band during the MC stage.

**Figure 7:**
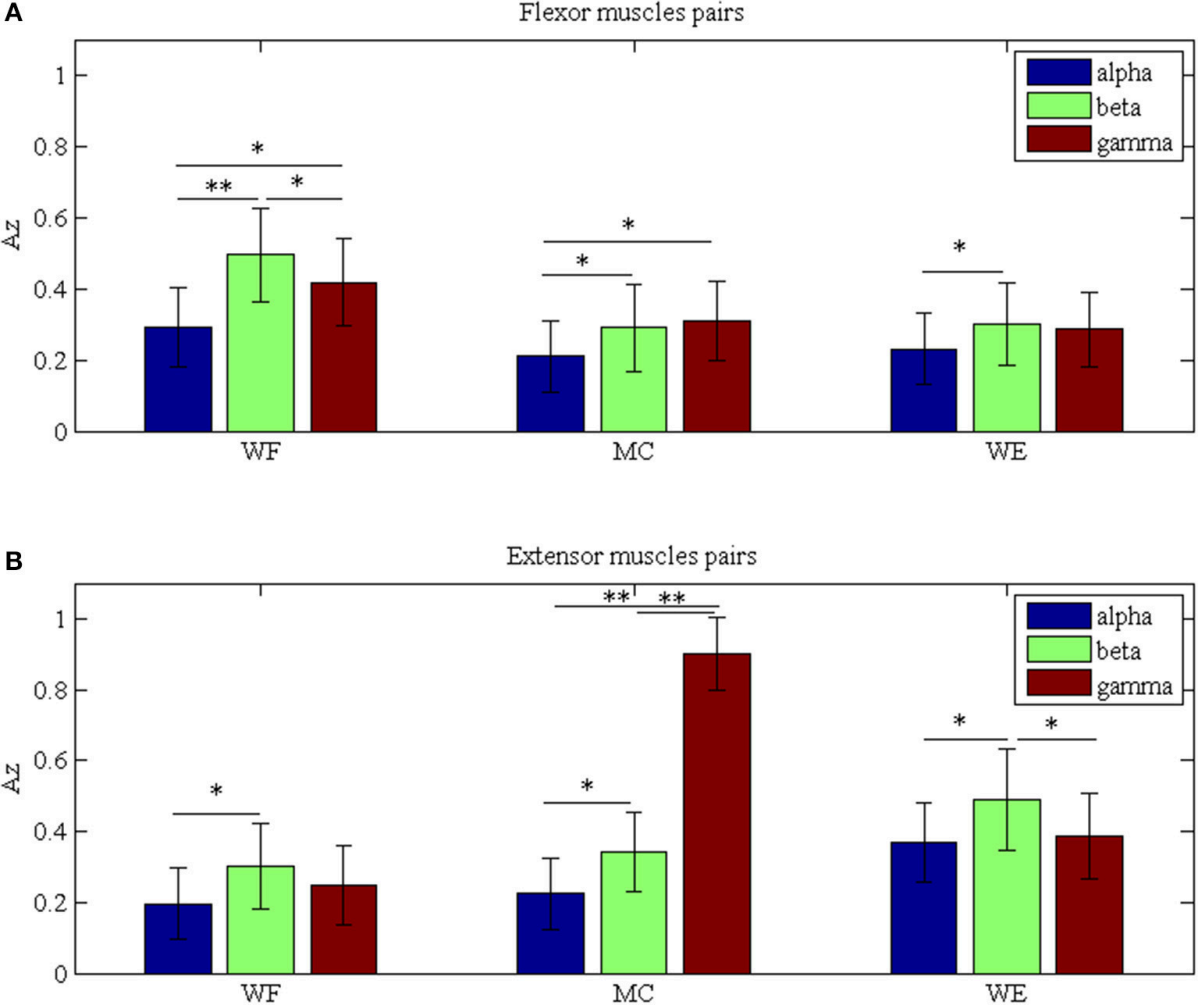
Grand averages of the *A*_*z*_ values in the three stages for **(A)** Flexor muscle pairs and **(B)** Extensor muscle pairs in the alpha, beta and gamma bands, respectively. We denoted the significance with the star mark. *p < 0.05 and **p < 0.01.

**Figure 8:**
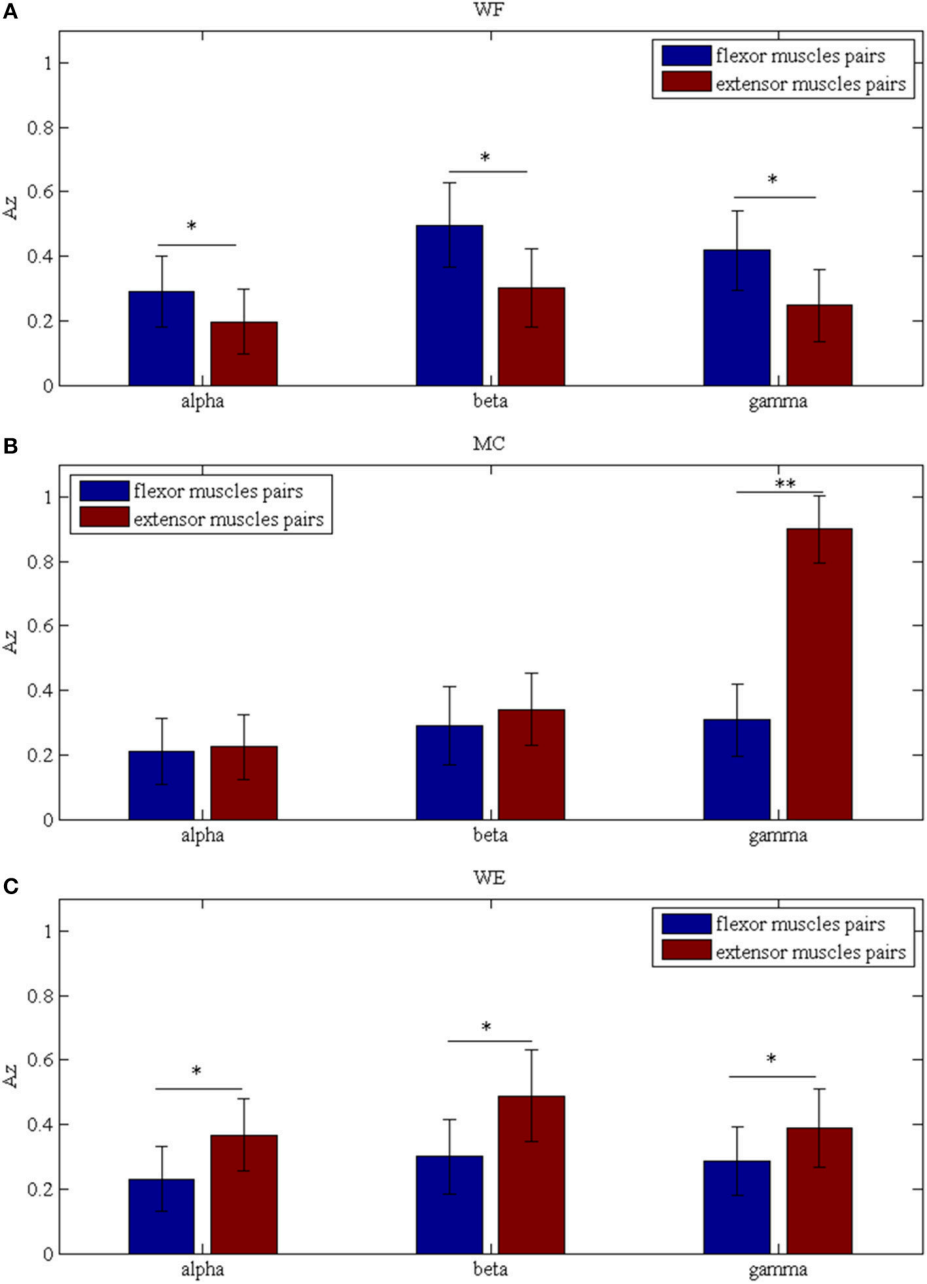
Comparison of the *A*_*z*_ values between the flexor and extensor muscle pairs in three bands during **(A)** WF stage, **(B)** MC stage, and **(C)** WE stage, respectively. We denoted the significance with the star mark. *p < 0.05 and **p < 0.01.

To investigate the differences between the flexor muscle pairs and extensor muscle pairs among three stages (WF, MC, and WE stage) at different frequency bands (alpha, beta and gamma), we performed three-way ANOVA. In our statistical analysis, we calculated the average values of the *A*_*Z*_ value for three flexor muscle pairs and extensor muscle pairs, respectively. To describe results intuitively, we performed normalization process. As Figure 7A was shown, multiple comparison showed that there was significant difference between each frequency band under each stage for flexor muscle pairs, except for between the beta and gamma bands under the MC stage [*F*_(2, 24)_ = 2.16, *p* = 0.104],between the alpha and gamma bands [*F*_(2, 24)_ = 2.46, *p* = 0.075] and between the beta and gamma bands under the WE stage [*F*_(2, 24)_ = 2.34, *p* = 0.086]. Similarly, Figure 7B also showed that there was significant difference between each frequency band under each stage for extensor muscle pairs, except for between the alpha and gamma bands [*F*_(2, 24)_ = 1.26, *p* = 0.112] and between the beta and gamma bands under WF stage [*F*_(2, 24)_ = 1.95, *p* = 0.102], between the alpha and gamma bands [*F*_(2, 24)_ = 0.67, *p* = 0.202] under WE stage. Obviously, the *A*_*Z*_ value in the beta band is the largest compared with the other bands during both WF and WE stages for flexor and extensor muscle pairs, and it is largest in the gamma band during MC stage for extensor muscle pairs. Additionally, we also compared the differences between the flexor muscle pairs and extensor muscle pairs among different stages as shown in Figure 8. In this figure, we can see that there was significant difference between flexor and extensor muscle pairs in three bands for each stage, except for in alpha [*F*_(2, 24)_ = 0.81, *p* = 0.197] and beta [*F*_(2, 24)_ = 0.91, *p* = 0.183] bands for MC stage. Compared to the flexor muscle pairs, the *A*_*Z*_ values of the extensor muscle pairs were lower in the WF stage, and higher in both MC and WE stages.

## Discussion

The significant contribution of this paper is the estimation of time-varying coherence amongst the synergistic muscles during the wrist movements based on the NMF and TFC methods. The results presented intermuscular coherence between each pairwise synergistic muscles. The intermuscular coherence showed higher *DC*_*xy*_(*l, f*) only in the beta band under WF stage and WE stages, and the coupling strength in the gamma band is the highest in MC stage. The time-varying coherence analysis within motor modules demonstrates the existence of correlated neural inputs for a specific group of synergistic muscles during the dynamic wrist tasks. These findings will be discussed from the point of neurophysiology.

### Muscle synergy mechanisms during wrist movement

Many previous studies reported that muscle synergy structure can reveal the co-activation pattern of muscles (Steele et al., 2013; Tang et al., 2015). In our study, we obtained consistent findings. Additionally, we found that muscle synergy analysis can extract synergistic muscles under specific movement. Our results showed three synergistic flexor pairs (FCR-PL, FCR-FDS, and PL-FDS) in the WF stage, whose weighting were higher in the module *W*_1_, as these muscles were recruited to support WF movement. And the time-varying coefficients *C*_1_ had a peak value in WF stage, which could reflect the contribution of synergistic muscles to the movement at this time period (Tang et al., 2015). It represents that the force exerted by the FCR, PL, and FDS muscles throughout this WF movement. We also found that three extensor pairs (ECU-ECR, ECU-B, and ECR-B) were activated in both the MC and WE stages. And the coefficient *C*_4_ has a peak value in MC and WE stage. We inferred that the active muscles associated with the wrist extension were recruited to support the wrist extension motion when the wrist was about to perform the wrist extension motion (MC stage), while the flexor muscles were in a relaxed state. It would suggest that the synergistic extensor muscles in the module *W*_4_ share common input from CNS. Huang et al. also found that the time-varying coefficient can be used to observe changes in the activation level of muscle-tendon units (MTUs) (Huang et al., 2016). These findings showed that muscle synergy was an effective model which could reveal the complex motor control mechanism. A possible interpretation to such observation could be that “the CNS of the mammal achieves movement by controlling muscle coordination” (Tresch et al., 1999) and further validate the hypothesis that the CNS employs modular control (Hug et al., 2010). Above all show that decomposition of the synergistic modules could be used to divide co-acting muscles and provide the basis for studying the motor control mechanism of the CNS.

### Intermuscular coherence amongst the muscles synergy analysis

Additionally, compared to the flexor muscle pairs, the intermuscular coherence of the extensor muscle pairs were lower in the WF stage, and higher in both MC and WE stages. From the view of results about synergy muscles, the flexor synergistic muscles played a key role in the WF stage but extensor synergistic muscles acted on the MC and WE stages. From an anatomical point of view, these flexor muscles are not independent and in fact share a function of WF in a big variety of tasks. The interpretation to such observation could be that these muscles are controlled by the same command from CNS in the WF stage. During the movement process, we assumed that the flexor muscles were controlled by a single instruction to prevent the muscle blending phenomenon, which was same to the conclusion in the other study (Roh et al., 2011). Likewise, the extensor muscles have same purpose in WE stage. In addition, the extensor muscles could recruit the neural information to perform WE task in MC stage due to the motor task from WF to WE stage. Therefore, the extensor muscles have same phenomenon in both MC and WE stages. Several researches have reported that intermuscular coherence exists in synergistic muscle pairs (De et al., 2015; Li et al., 2016). The interpretation maybe that the synergy mainly related to force production and the intermuscular synchronization occurs when functional forces are produced. Therefore, the contribution level of synergistic muscle is related to the coupling strength. We concluded that the coding patterns of neural control motors used to produce various movements were different among the three time periods.

### Beta and gamma modulation in the motor control

Further analysis showed that the coherence between the flexor muscle pairs was mainly observed in the beta band (15–35 Hz) during the WF stage, and that within the extensor muscle pairs was also observed in the beta band during the WE stage, which was similar to the conclusion that the movement control information transmitted by neurons was primarily in the beta frequency band (Norton et al., 2003). Beta band oscillations in muscle activity are thought to arise due to the result of a common cortical drive from the CNS (Fisher et al., 2012). Many researches on this function suggested that they could be used for sensorimotor processing or calibration (Baker et al., 1997) or promote stable motor output (Pogosyan et al., 2009). Reyes (Reyes et al., 2017) suggested that beta band cortical drive essentially reflects the dimensionality of cortical commands. This observation in our work is possibly related to the wrist stabilizing function of these muscles during the steady-state tasks. However, the intermuscular coherence amongst the extensor muscle pairs mainly on gamma band during the MC stage, which is the mainly related to force production. This observation in the gamma band is in line with previous studies found that the intermuscular coherence strength shifted toward the gamma frequency band for the rapid integration of information produced by the CNS (Omlor et al., 2007). The CNS could regulate the control mode of the related muscles to increase the control strength of the upper limb, which enhanced synchronous coupling, such that the intermuscular coherence strength in the gamma band increased (Patino et al., 2008). Gibbs et al. (1995) found where a common drive was detected with lower limb muscle pairs, the degree of synchrony was significantly larger during balancing task than during either lying or standing. The intermuscular coherence in the gamma band could be interpreted by the need of rapidly integrating information and producing the corresponding motor commands during the dynamic motor task, and it might thus reflect a corticomuscular coupling within the same bands between cortical activity and the single muscles.

## Conclusion

In this paper, we analyzed the time-varying coherence characteristics based on the NMF-TFC method during the wrist movement in healthy individuals. The results about the significant intermuscular coherence between each pairwise synergistic muscles shows us that synchronous oscillations contribute to the modulation of module structure. The stronger coupling in the beta band under the WF and WE stages indicates that beta band contributes to containing the steady state, and the intermuscular coherence in the gamma band during the transition from WF to WE stage shows that gamma band is related to the dynamic force output. These results demonstrated the time-varying mechanisms of the synergistic modulation and synchronous oscillation in motor-control system. This study contributes to expanded mechanism for motor control and providing an effective approach for our future research.

## Author contributions

PX, GH, and WY were responsible for the design of the research. WQ and XL were in charge of collection and analyzing the EMG data. XC provided the physiological mechanism for this discussion. PX, GH, and WY drafted and integrated the manuscript in progress. YD offered the comments for the revised paper. All authors have read and approved the final manuscript.

### Conflict of interest statement

The authors declare that the research was conducted in the absence of any commercial or financial relationships that could be construed as a potential conflict of interest.
