# Supplementary material for: Estimation of Time-Varying Coherence Amongst Synergistic Muscles During Wrist Movements
Source: Front Neurosci. 2018 Aug 7;12:537. doi: 10.3389/fnins.2018.00537 (PMC6090894; doi:10.3389/fnins.2018.00537)
Supplement: Supplementary file 1 [file Presentation_1.PDF]

# Appendix

**Introduction of Delsys system**

The Delsys system is a wirelessly transmitting physiological monitoring system produced by DELSYS INC, which is equipped with electrodes for continuously recording EMG signals. The system is as shown in Figure 1.

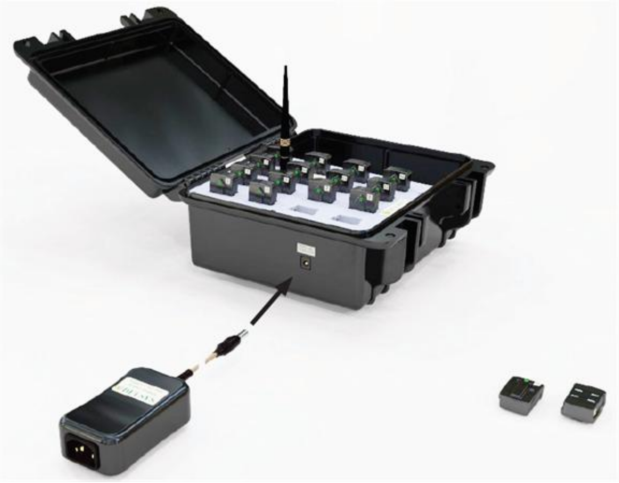

Figure 1. The diagram of the Delsys system.

There are two types of sensors with different sampling frequency to record the EMG signals in the Trigno™ Wireless EMG system. One type of sensor with nine degrees of freedom is used with the Trigno™ Wireless EMG system designed to detect surface EMG signals with additional movement-sensing modalities reporting Acceleration (g), rotation (deg/s) and magnetic field (uT) in three orthogonal axis, whose sampling frequency is 1112 Hz. The EMG signal detection features of this sensor preserve all the fundamental characteristics of the standard sensor with three degrees of freedom. The another type of sensor is with three degrees of freedom reporting Acceleration (g) in three orthogonal axis, whose sampling frequency is 1927 Hz. The two types of sensors both have a 10mm inter-electrode distance with full bandwidth sEMG signal detection from 0-450 Hz to ensure consistency, repeatability and data quality. In our experiment, the sensor outputs a digital stream of eight data channels, all time-synchronized to each other and to all other active sensors in the system. The two types of sensors constructed with a differential circuit and a reference circuit is equal to two dry electrodes to avoid an additional electrode or gel to paste the electrodes. They all have four silver rod contacts used to detect EMG signals on the skin surface. The sensor is as shown in Figure 2.

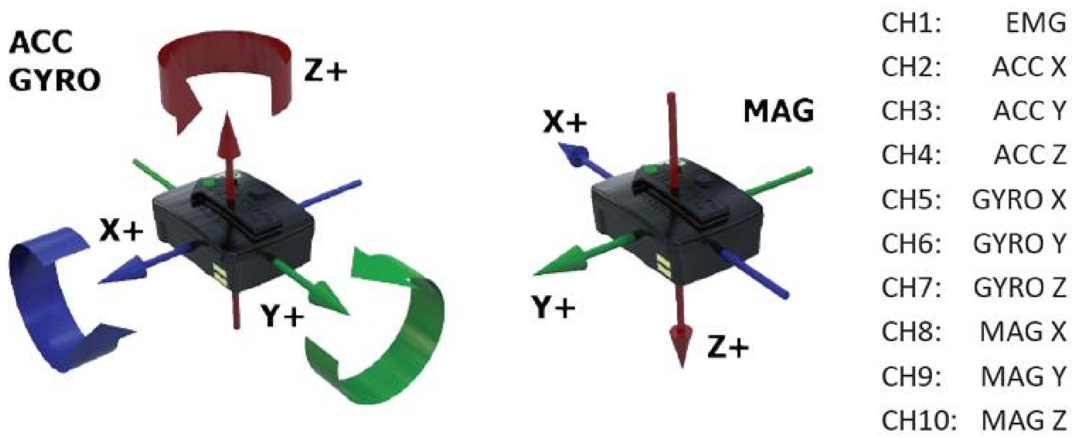

Figure 2. The diagram of the sensors. Left-axial definition of the accelerometer and gyroscope output signals. Right- definition for the magnetometer outputs.
